# Prospective in-depth analysis of anaesthetic management of spontaneous ventilation VATS for lung cancer resection: a matched pairs comparison to intubated VATS

**DOI:** 10.1186/s12871-025-03027-9

**Published:** 2025-04-16

**Authors:** Lorenz L. Mihatsch, Anastasia Huber, Sandra Weiland, Patrick Friederich

**Affiliations:** 1 TUM School of Medicine and Health, Technical University of Munich, TUM University Hospital, Munich, Germany; 2 Department of Anaesthesiology, Intensive Care Medicine, Pain Therapy, München Klinik Bogenhausen, Technical University of Munich, Munich, Germany; 3 Institute for Medical Information Processing, Biometry, and Epidemiology, Ludwig- Maximilians-Universität, Munich, Germany

**Keywords:** SV-VATS, Thoracic surgery, Thoracic anaesthesia, I-VATS, Double-lumen tube

## Abstract

**Background:**

Spontaneous ventilation video-assisted thoracoscopic surgery (SV-VATS) has been propagated for nearly two decades without a prospective in-depth analysis of anaesthetic management and anaesthetic processing times. This would be important as anaesthetic management of SV-VATS imposes fundamental changes to standards in thoracic anaesthesia and may increase anaesthetic risks. Therefore, this study aimed to provide such in-depth analysis and compare the results to data from matched intubated VATS (I-VATS) patients. 3D-reconstruction of bronchial airways helped to estimate the risk reduction by avoiding double-lumen tube (DLT) intubation according to common selection methods in SV-VATS patients.

**Methods:**

SV-VATS patients receiving anatomical (*N* = 22) and non-anatomical (*N* = 16) lung cancer resections were prospectively enrolled. A retrospective I-VATS control cohort (*N* = 76) allowed for a 2:1 propensity score matching. DLT sizes necessary for SV-VATS patients according to common selection methods were evaluated by 3D-reconstruction of the left main bronchus and the ≥ 1 mm criterion.

**Results:**

SV-VATS patients required substantially less propofol dosage (*P* < 0.001) with an increase in variability of drug dosing (*P* < 0.001) and higher BIS values (*P* < 0.001) as compared to I-VATS patients. SV-VATS lead to higher variability in respiratory parameters (*P* < 0.001) with less driving pressure (*P* < 0.001) and similar mean tidal volumes, oxygenation, and hemodynamic parameters compared to I-VATS. Spontaneous ventilation was achieved by allowing for permissive hypercapnia and respiratory acidosis. Anaesthetic processing time was reduced by 7 min (*P* < 0.001). 5–10% of female and 5% of male patients would have received a DLT larger than their bronchial airway.

**Conclusions:**

Our study provides the first prospective quantitative in-depth analysis of a standardised anaesthetic management regime for SV-VATS, including anaesthetic processing times. Respiratory parameters during SV-VATS are compatible with reduced mechanical power as compared to patients undergoing I-VATS. The anaesthetic management regime reduced the risk of airway damage imposed by choosing too-large DLTs in up to 10% of patients without compromising oxygenation and hemodynamic stability. Changes in anaesthetic processing time by 7 min would not allow for a higher caseload of SV-VATS for lung cancer surgery.

**Clinical trial number:**

Not applicable.

**Supplementary information:**

The online version contains supplementary material available at 10.1186/s12871-025-03027-9.

## Background

Spontaneous ventilated video-assisted thoracoscopic surgery (SV-VATS) has been established for thoracic surgical procedures ranging from pleurectomy to pneumonectomy [1–10]. It has been proposed as superior to intubated VATS (I-VATS) in avoiding complications from intubation [11], muscle relaxation, and one-lung ventilation (OLV) [2, 7, 9, 12–16]. Despite being propagated and extensively described by thoracic surgeons for nearly two decades [3, 6, 17, 18] randomised controlled (multicentre) trials are still lacking. Beyond the surgical technique, a detailed data-driven analysis of anaesthetic management is missing [7]. This is astonishing as SV-VATS requires fundamental changes to well-established standards in thoracic anaesthesia concepts [7] and may impose additional risk to these patients [9, 19].

Several different anaesthetic techniques have been suggested for SV-VATS over the last two decades ranging from epidural anaesthesia or paravertebral nerve block combined or not with analgosedation and intrathoracic vagal blockade to general anaesthesia combined or not with intrathoracic vagal blockade allowing for spontaneous ventilation aided by basic facemask, non-invasive laryngeal mask airway or invasive double-lumen tube [7, 9, 20, 21]. Detailed clinical data on hemodynamic and ventilatory management, blood gas analyses, as well as anaesthetic drug application with target concentrations of the effect compartment during SV-VATS, however, only using a laryngeal mask instead of endotracheal intubation have not been provided so far. A recent retrospective analysis comparing non-intubated VATS (NI-VATS) patients using facemasks to I-VATS patients did not find significantly more desaturation events in NI-VATS patients; however, NI-VATS patients had significantly greater end-tidal CO2 during anaesthesia, and the group of I-VATS had significantly more sore throats after anaesthesia [16]. Furthermore, although anaesthetic management of SV-VATS has been promoted as less time-consuming [3], it remains unclear whether this proposed benefit translates into a higher caseload in real-world clinical settings compared to I-VATS.

Multiple meta-analyses have reported that NI-VATS is associated with a lower overall rate of postoperative complications compared to I-VATS [9, 14, 22]. This advantage is likely due to the avoidance of mechanical ventilation-related lung injury, residual neuromuscular blockade, and especially intubation-associated airway trauma with double-lumen tubes (DLT) [14].

A very common anaesthetic concept for SV-VATS constitutes airway management with a laryngeal mask, hypnosis with propofol, and analgesia with a combination of opioids, intercostal block, and vagal nerve blockade [7]. Therefore, the present analysis aims to provide the first detailed analysis of this defined and standardised anaesthetic SV-VATS regime, including anaesthetic processing times and 3D-reconstruction of the bronchial airways for lung cancer resection in order to estimate the DLT risk in this specific population. Matched pairs analysis was applied to compare anatomic and non-anatomic SV-VATS to I-VATS patients.

## Methods

From 11/2018 to 10/2021, 36 patients were scheduled for SV-VATS (22 for anatomical and 16 for non-anatomical resection) (Table 1) at Munich Clinic Bogenhausen, an academic teaching hospital of the Technical University of Munich, Germany. For the retrospective I-VATS group, a total of 218 patients from the time period 02/2017–12/2019 were considered. However, only 187 (95 anatomical and 92 non-anatomical resections) patients were included in the matching process, as 31 patients had to be excluded due to lung volume-reducing previous surgeries. Using a 1:2 propensity score matching, 76 I-VATS patients (44 receiving anatomical and 32 receiving non-anatomical resection) (Table 2) were matched as the control groups as described below.

**Table 1:** Types of anatomical VATS performed

	SV-VATS, *N* = 22		I-VATS, *N* = 44	
	Right	Left	Right	Left
Lingula resection	-	1	-	-
Segment resection	1	-	1	-
Upper lobectomy	-	-	12	7
Middle lobectomy	5	-	2	-
Lower lobectomy	6	7	10	9
Upper bilobectomy	-	-	2	-
Lower bilobectomy	1	-	1	-
Pneumonectomy	1	-	-	-
Total	14	8	28	16

**Table 2:** Types of non-anatomical VATS performed

		SV-VATS, *N* = 16		I-VATS, *N* = 44	
		Right	Left	Right	Left
Location	Upper lobe	3	2	10	2
	Middle lobe	1	-	1	-
	Lower lobe	6	3	8	5
	Middle + lower lobe	-	-	2	-
	Upper + lower lobe	1	-	-	3
	Total	11	5	21	11
# of wedges	1	8	4	15	6
	2	2	1	5	3
	3	1	-	1	2
	Total	11	5	21	11
Lymphad-nectomy	yes	3	1	12	2
	no	8	4	11	7
	Total	11	5	23	9
Pleurec-tomy	yes	1	-	3	-
	no	10	5	17	12
	Total	11	5	20	12

**Fig. 1:**
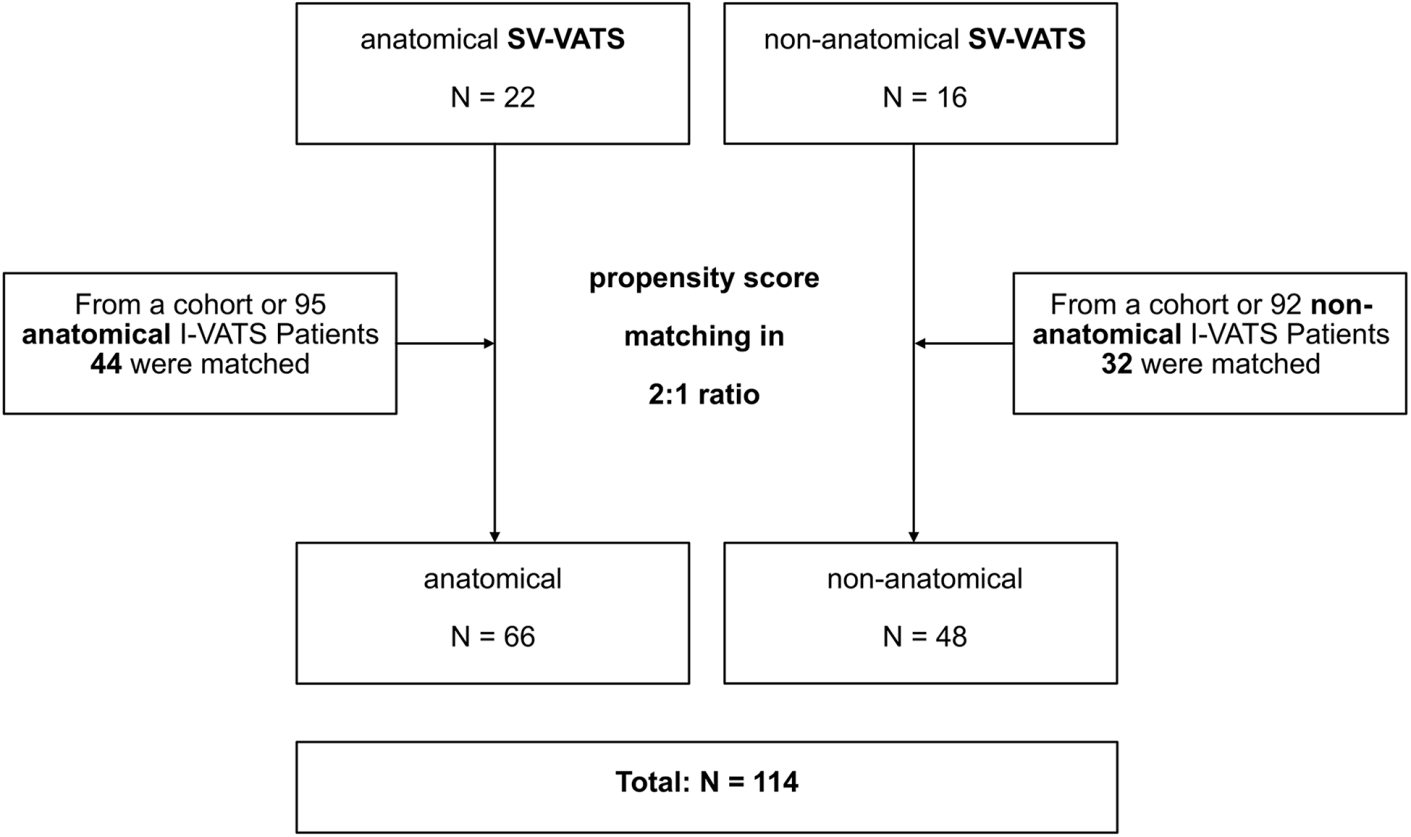
Patient flow diagram. Propensity score matching was based on 13 (anatomical resection 12) pre-operative variables: age, sex, height, weight, body mass index (BMI), forced vital capacity (FVC), forced expiratory volume (FEV1), FEV1/FVC, total lung capacity (TLC), DLCO-SB (only anatomical resections), paO2, paCO2, haemoglobin

In total, 114 patients were included (Fig. 1). Inclusion criteria were (i) thoracic surgery in VATS, (ii) age ≥ 18 years, and (iii) written informed consent. Exclusion criteria were (i) pathology of the upper airway, such as laryngeal or pharyngeal swelling, tumours, or likewise, (ii) BMI ≥ 35, (iii) intracranial pathology, and (iv) coagulation disorder. A STROBE statement can be found in Supplementary Material M1.

### Ethics

Patients receiving I-VATS were enrolled in agreement with the Bavarian Hospital Act (BayKrG, Art. 27). All patients receiving SV-VATS gave written informed consent to participate in the study prior to inclusion. Ethical approval was waived by the ethical committee of the Bayerische Landesärztekammer (2020 − 1041) since all data collected were routine and observational in nature and, thus, fall under the provisions of the Bavarian Hospital Act (BayKrG, Art. 27).

### Anaesthetic management

All patients were routinely instrumented with 16- and 18-gauge peripheral venous access and an arterial blood pressure catheter. Anaesthetic management of patients undergoing SV-VATS included EEG (BIS^®^, Medtronic AG, Meerbusch, Germany) monitored target-controlled infusion (Agilia Injectomat TIVA^®^, Fresenius AG, Bad Homburg, Germany) of propofol (Marsh Model [23]) and remifentanil (Minto Model [24]) with laryngeal mask airway (AmbuAuraGain^®^, Ambu GmbH, Bad Nauheim, Germany) after lidocaine 4% inhalation and pharyngeal lidocaine 4% instillation, intercostal nerve block and intrathoracic vagal blockade (bupivacaine 0.5%), andpressure support ventilation (Zeus^®^ or Perseus^®^, Dräger AG, Lübeck, Germany).

Anaesthetic management for I-VATS was left to the discretion of the anaesthesiologist. It included target-controlled infusion (Agilia Injectomat TIVA^®^, Fresenius AG, Bad Homburg, Germany) of propofol (Marsh Model [23]) and bolus application of fentanyl, with or without the combination of volatile anaesthetics (desflurane or sevoflurane), muscle relaxation with atracurium, EEG monitoring (BIS^®^, Medtronic AG, Meerbusch, Germany), and intubation with a double-lumen tube (EPSA^®^ double-lumen bronchial tube, Electroplast S.A., Montevideo, Uruguay) followed by controlled two-lung and one-lung ventilation (OLV) (Zeus^®^ or Perseus^®^, Dräger AG, Lübeck, Germany) as appropriate during the operation. Of the I-VATS patients receiving anatomical lung resections, 8 were subjected to propofol, and 36 to propofol and a volatile anaesthetic. Of the I-VATS patients receiving non-anatomical resections, 7 were subjected to propofol, and 25 to propofol and a volatile anaesthetic.

### Propensity score matching (PSM)

Using a binary logistic regression model based on 13 preoperative covariables (*age*,* sex*,* height*,* weight*,* body mass index (BMI)*,* forced vital capacity (FVC)*,* forced expiratory volume (FEV1)*,* FEV1/FVC*,* total lung capacity (TLC)*,* lung diffusion capacity for CO (DLCO-SB; only anatomical resections)*,* paO2*,* paCO2*,* haemoglobin (Hb)*) and a retrospective pool of 95 anatomical and 92 non-anatomical I-VATS patients, the propensity score was calculated for each individual of the retrospective pool. The propensity score indicates the “likelihood” for an individual of being subjected to an SV-VATS instead of an I-VATS. An optimal pair matching algorithm [25] matched two I-VATS patients of the retrospective pool for each SV-VATS patient based on similar propensity scores. The 2:1 matching was performed separately for patients with anatomical and non-anatomical resections. Standardised mean differences (SMD) between SV-VATS and I-VATS are substantially smaller after matching for all variables except for FEV1. There is no significant difference in any variable after matching (Suppl. Table S1). This approach enhances the internal validity of our findings and makes the results more applicable to real-world clinical settings, simulating an RCT-like environment without the need for randomisation [26–28].

### Sample size calculation

For an independent two-sample, two-sided t-test with a level of significance of α = 0.05, a power of (1-β) = 0.8 for the group of anatomical resections (SV-VATS: *N* = 16; I-VATS: *N* = 32) the minimal detectable effect size is Cohen’s d = 1.45 and for non-anatomical resection (SV-VATS: *N* = 22; I-VATS: *N* = 44) Cohen’s d = 0.62 Correspondingly, for a non-parametric Wilcoxon Rank Sum test, the minimum detectable effect size was *r* = 0.40 for anatomical and *r* = 0.34 for non-anatomical resection.

### Data collection

SV-VATS were assessed prospectively, and I-VATS patients retrospectively. All patients received a spirometry and blood gas analysis (BGA) before surgery. Intra-operative variables for haemodynamic and ventilation were recorded every 15 min. BGA were performed at the discretion of the anaesthesiologist during OLV.

### 3D-Reconstruction and DLT-Selection

Based on 2D-CT scans the diameter of the left main bronchus of the SV-VATS patients’ airways were reconstructed in 3D using a standard 3D reconstruction software (IntelliSpace Portal 12, Philips, Amsterdam, Netherlands) and as described in our previous work [11]. The software automatically calculates the perfect cross-section of the left bronchial diameter. Diameters were calculated for each slice, perpendicular to the bronchial wall. The slice 10 mm distal of the carina was used, where the bronchial cuff of the DLT approximately comes at rest. The diameter of the bronchial branches of EPSA-DLTs (EPSA^®^ double-lumen bronchial tube, Electroplast S.A., Montevideo, Uruguay) was also retrieved from our previous work [11]. The selection methods based on demographic variable, i.e., sex and body height [29], the tracheal diameter on a chest X-ray [30], and the bronchial diameter on a 2D-chest CT [31] are described elsewhere [11].

### Statistical analysis

Perioperative numeric variables were tested using a t-test or non-parametric Wilcoxon rank sum test, and categorical variables using a χ^2^-test and Fisher’s exact test as appropriate. Intra-individual means, standard deviations (SD), and the coefficients of variation respecting the repeated measurements were calculated for haemodynamic and respiratory parameters, which were assessed every 15 min intraoperatively. Blood gas variables were not recorded in the same frequency in SV-VATS and I-VATS and were thus analysed by group. Thus, we averaged the BGA per individual across the time of OLV and calculated SD from the averaged values.

Time intervals were compared using log-rank tests. To assess if the time of recovery from anaesthesia depended on the incision-suture time, a Cox proportional hazard (Cox-PH) model was used with incision–suture time and VATS group as covariables. The contribution of incision-suture time was tested for significance using a likelihood ratio test (LRT).

To correlate the incision-suture time with the intra-individual coefficients of variation of propofol, the three groups of SV-VATS, I-VATS with propofol, and I-VATS without propofol were adjusted for by calculating partial correlations [32].

*P*-values are corrected for multiple testing using Bonferroni’s method when appropriate. A *P*-value < 0.05 was considered statistically significant.

## Results

### Baseline characteristics before surgery

Patients’ characteristics, preoperative spirometry, and blood gas parameters are summarised in Table 3. There was no significant difference between the variables for patients receiving anatomical or non-anatomical resections. Thus, the intervention and control groups were balanced before surgery.

**Table 3:** Patient characteristics and pre-surgical parameters after propensity score matching. Numeric variables are given as mean ± sd. Categorical variables as absolute count *n/N* (%)

	Anatomical Resections			Non-Anatomical Resections		
	**SV-VATS**, *N* = 22	**I-VATS**, *N* = 44	*P*– Value^3^	**SV-VATS**, *N* = 16	**I-VATS**, *N* = 32	*P*– Value^3^
Sex - male^1^	7/22 (32%)	14/44 (32%)	> 0.999	9/16 (56%)	19/32 (59%)	> 0.999
Age [years]^2^	69 ± 9.5	68 ± 10.4	0.714	58 ± 16.5	61 ± 14.4	0.582
Height [cm]^2^	167 ± 8.4	167 ± 9.4	0.682	175 ± 10.8	174 ± 9.9	0.757
Weight [kg]^2^	69.2 ± 11.0	69.3 ± 13.7	0.995	77.8 ± 17.5	78.0 ± 15.3	0.955
BMI [kg/m^2^]^2^	25.0 ± 3.5	24.7 ± 4.8	0.846	25.4 ± 4.8	25.9 ± 4.4	0.752
RCRI:^1,4^ 0	16/22 (73%)	37/44 (84%)	0.271	16/16 (100%)	27/32 (84%)	0.425
1	5/22 (23%)	7/44 (16%)		0/16 (0%)	2/32 (6%)	
2	1/22 (5%)	0/44 (0.0%)		0/16 (0%)	2/32 (6%)	
3	-	-		0/16 (0%)	1/32 (3%)	
TLC [l]^2^	5.58 ± 1.10	5.68 ± 1.20	0.734	6.85 ± 1.67	6.57 ± 1.71	0.602
VC [l]^2^	2.91 ± 0.76	2.97 ± 0.99	0.796	3.78 ± 1.55	3.60 ± 1.10	0.639
FEV1 [l]^2^	2.18 ± 0.59	2.24 ± 0.75	0.746	3.02 ± 1.27	2.85 ± 0.91	0.587
FVC [l]^2^	2.84 ± 0.76	2.93 ± 1.04	0.726	3.82 ± 1.39	3.61 ± 1.21	0.596
FEV1 / FVC^2^	0.79 ± 0.07	0.78 ± 0.11	0.741	0.79 ± 0.08	0.80 ± 0.09	0.821
FRC [l]^2^	3.31 ± 0.95	3.54 ± 0.90	0.333	3.77 ± 1.09	4.01 ± 1.29	0.541
PEF [l/s]^2^	5.39 ± 1.64	5.60 ± 1.88	0.656	7.43 ± 2.90	6.95 ± 2.05	0.523
MEF50 [l/s]^2^	2.50 ± 0.95	2.59 ± 1.42	0.806	3.67 ± 2.09	3.50 ± 1.42	0.762
sRtot [kPa×s/l]^2^	1.09 ± 0.43	1.32 ± 0.59	0.112	1.18 ± 0.64	1.15 ± 0.49	0.865
DLCO SB^2,5^	6.61 ± 1.36	6.57 ± 1.99	0.929	-	-	-
pH^2^	7.43 ± 0.02	7.44 ± 0.03	0.604	7.44 ± 0.02	7.44 ± 0.03	0.771
paCO2 [mmHg]^2^	34.31 ± 3.84	33.85 ± 4.12	0.662	33.89 ± 3.06	33.16 ± 3.17	0.455
paO2 [mmHg]^2^	77.25 ± 7.84	77.27 ± 9.47	0.994	78.82 ± 10.03	79.21 ± 8.84	0.891
AaDO2 [mmHg]^2^	28.40 ± 7.25	28.01 ± 9.22	0.865	27.10 ± 9.94	29.65 ± 22.58	0.688
SpO2 [%]^2^	95.80 ± 1.63	95.59 ± 1.74	0.601	96.34 ± 1.94	96.06 ± 1.25	0.561
Hb [mg/dl]^2^	12.36 ± 1.36	12.51 ± 1.83	0.739	11.95 ± 1.39	11.71 ± 1.33	0.569

^1^*n/N* (%); ^2^ mean ± SD; ^3^ χ^2^-tests, Fisher-Exact-tests, Wilcoxon Rank Sum tests were used as appropriate; ^4^ Values may not sum to 100% due to rounding effects; ^5^ DLCO SB was not measured systematically for patients receiving non-anatomical resections

Abbreviations: BMI– body mass index, RCRI– revised cardiac risk index, TLC– total lung capacity, VC– vital capacity, FEV1– forced expiratory volume in 1 s, FVC– forced vital capacity, FRC– functional residual capacity, PEF– peak expiratory flow, MEF50– maximal expiratory flow at 50% of FVC, sRtot– total specific resistance, DLCO-SB– lung diffusion capacity for CO, Hb– haemoglobin

### Anaesthetic management

**Fig. 2:**
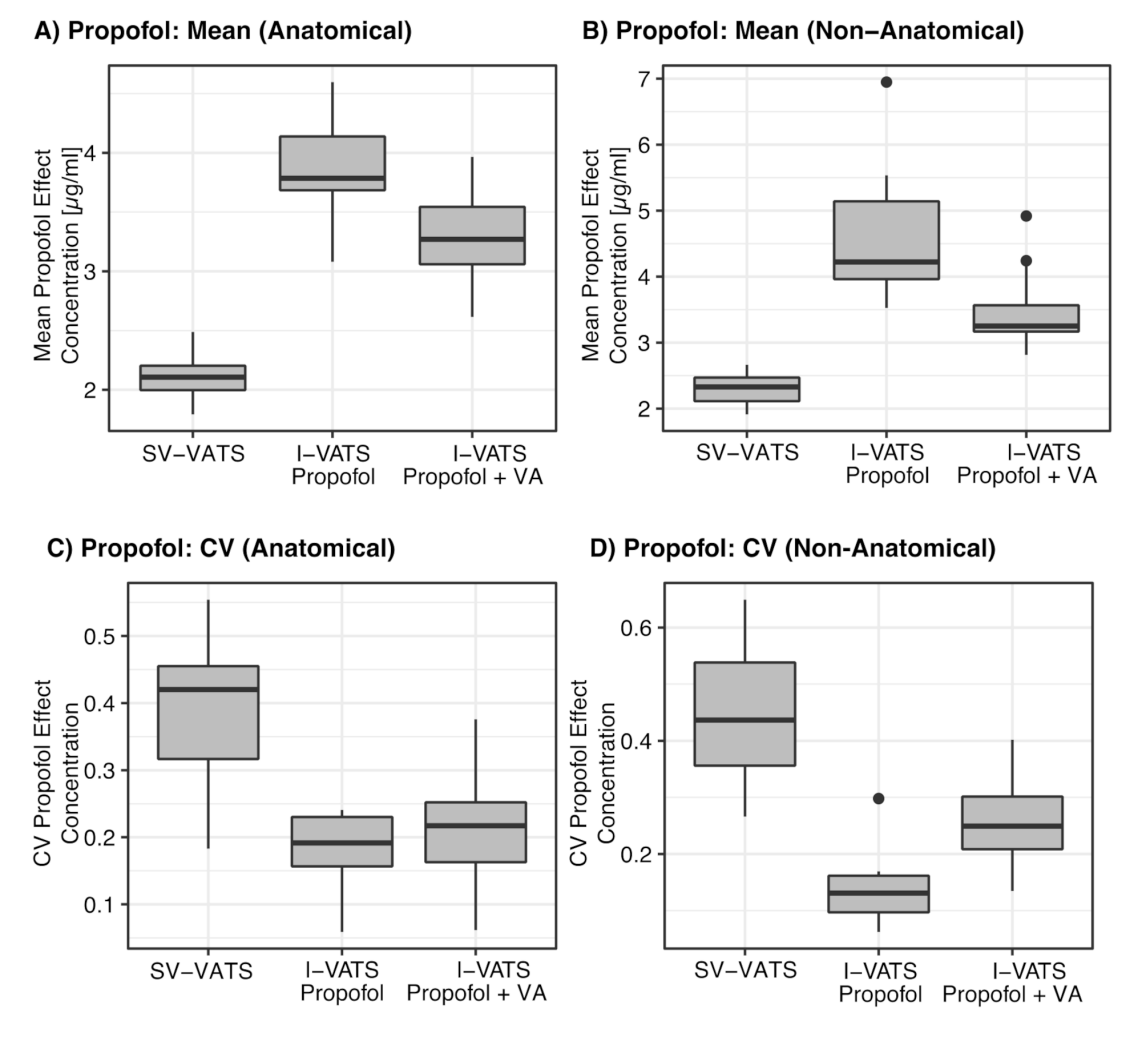
Mean of propofol target concentrations for the effect compartment during anatomical (**A**) and non-anatomical VATS (**B**). Coefficient of variation (CV) of propofol TCI of anatomic (**C**) and non-anatomic surgery (**D**). Of the patients receiving anatomical lung resections, 22 were subjected to SV-VATS, 8 to I-VATS with propofol, and 36 to I-VATS with propofol and volatile anaesthetic (VA). Of the patients receiving non-anatomical resections, 16 were subjected to SV-VATS, 7 to I-VATS with propofol, and 25 to I-VATS with propofol and volatile anaesthetic

SV-VATS patients received significantly lower target concentrations for the effect compartment of propofol than I-VATS patients (anatomical resections: SV-VATS 2.1 ± 0.8 µg/ml, I-VATS propofol 3.9 ± 0.7 µg/ml, I-VATS propofol and volatile anaesthetic 3.3 ± 0.7 µg/ml, ANOVA *P* < 0.001; non-anatomical resection: SV-VATS 2.3 ± 1.0 µg/ml, I-VATS propofol 4.7 ± 0.7 µg/ml, I-VATS propofol and volatile anaesthetic 3.5 ± 0.9 µg/ml, ANOVA *P* < 0.001, Fig. 2A, B). The respective coefficients of variations for the target concentrations of propofol were significantly higher in SV-VATS patients (anatomical resections: SV-VATS coefficients of variation 0.44, I-VATS propofol coefficients of variation 0.14, I-VATS propofol and volatile anaesthetic coefficients of variation 0.26, Kruskal-Wallis *P* < 0.001; non-anatomical resection: SV-VATS coefficients of variation 0.44, I-VATS propofol coefficients of variation 0.14, I-VATS propofol and volatile anaesthetic coefficients of variation 0.26, Kruskal-Wallis *P* < 0.001, Fig. 2C, D).

Additionally, anatomical SV-VATS patients received remifentanil with a target concentration for the effect compartment of 2.07 ± 0.8 ng/ml (coefficients of variation 0.38), and non-anatomical SV-VATS patients of 2.14 ± 0.9 ng/ml (coefficients of variation 0.45). Opposingly, I-VATS patients received fentanyl at the discretion of the anaesthesiologist, anatomical 0.37 ± 0.1 mg and non-anatomical 0.34 ± 0.1 mg.

For anatomical patients, BIS values were 51.4 ± 6.0 for SV-VATS patients, 35.6 ± 2.8 for I-VATS patients receiving propofol, and 31.6 ± 5.0 for I-VATS patients receiving propofol and a volatile anaesthetic (Kruskal-Wallis Rank Sum test *P* < 0.001). For non-anatomical patients, they were 53.4 ± 4.8 for SV-VATS patients, 28.9 ± 0.6 for I-VATS patients receiving propofol, and 28.9 ± 0.6 for I-VATS patients receiving propofol and a volatile anaesthetic (Kruskal-Wallis Rank Sum test *P* < 0.001).

### Haemodynamic parameters

Mean haemodynamic variables did not significantly differ between SV-VATS and I-VATS patients receiving anatomical or non-anatomical resections (Table 4). The cumulative noradrenaline requirement was also not significantly different between SV-VATS and I-VATS patients (anatomical resections: SV-VATS median 15.0 µg/kg (IQR: 11.9–27.8 µg/kg), I-VATS median 16.1 µg/kg (IQR: 6.5–30.3 µg/kg), Wilcoxon rank sum test *P* = 0.865; non-anatomical resection: SV-VATS median 5.2 µg/kg (IQR: 2.4–9.4 µg/kg), I-VATS median 4.1 µg/kg (IQR: 0.0–8.4 µg/kg), Wilcoxon rank sum test *P* = 0.572). The coefficients of variation of the haemodynamic variables were not significantly different between SV-VATS and I-VATS patients. There was no significant difference between the haemodynamic parameters of I-VATS patients with and without volatile anaesthetic in addition to propofol (Suppl. Table S2).

**Table 4:** Intraindividual means and SD of intra-operative parameters are compared between SV-VATS and I-VATS patients. *P*-value are bonferroni corrected

	Anatomical Resections			Non-Anatomical Resections		
	**SV-VATS**, *N* = 22	**I-VATS**, *N* = 44	*P*– Value^3^	**SV-VATS**, *N* = 16	**I-VATS**, *N* = 32	*P*– Value^3^
**Hemodynamic Variables**						
Heart rate [1/min]^1^	70 ± 6.9	63 ± 5.6	0.199	70.6 ± 6.7	65.9 ± 5.6	> 0.999
Syst. BP [mmHg]^1^	109 ± 12.0	112 ± 9.9	> 0.999	113.2 ± 11.4	109.4 ± 8.8	> 0.999
Dia. BP [mmHg]^1^	55 ± 5.9	59 ± 5.4	0.767	58.1 ± 6.1	59.6 ± 5.7	> 0.999
MAP [mmHg]^1^	73 ± 7.7	77 ± 6.2	> 0.999	76.5 ± 7.1	76.2 ± 6.2	> 0.999
**Ventilation Setting**						
Vent. Freq. [1/min]^1^	12 ± 4.0	14 ± 1.1	0.152	12.3 ± 4.2	13.8 ± 1.5	0.441
etCO2 [mmHg]^1^	50.9 ± 5.9	35.3 ± 2.7	**< 0.001**	48.8 ± 5.0	34.5 ± 3.0	**< 0.001**
SpO2 [%]^1^	97 ± 1.7	99 ± 1.5	**0.003**	98.2 ± 1.3	98.4 ± 1.9	> 0.999
TV [ml/kg_PBW_]^1^	5.0 ± 1.8	5.8 ± 0.9	0.572	4.5 ± 1.5	5.3 ± 1.0	0.154
PEEP [mbar]^1^	3.0 ± 1.1	5.8 ± 1.2	**< 0.001**	3.4 ± 1.4	6.1 ± 1.0	**< 0.001**
Pmean– PEEP / ΔP [mbar]^1,4^	1.2 ± 1.0	11.9 ± 1.4	**< 0.001**	1.1 ± 0.5	11.6 ± 1.6	**< 0.001**
Ppeak– PEEP / ΔP [mbar]^1,4^	4.3 ± 1.9	11.9 ± 1.4	**< 0.001**	5.3 ± 1.6	11.6 ± 1.6	**< 0.001**
**Blood Gas Analysis**						
pH^2^	7.21 ± 0.06	7.34 ± 0.07	**< 0.001**	7.25 ± 0.04	7.37 ± 0.05	**< 0.001**
paCO2 [mmHg]^2^	60.6 ± 10.7	40.9 ± 10.7	**< 0.001**	55.6 ± 5.3	38.6 ± 7.5	**< 0.001**
paO2 [mmHg]^2^	163.3 ± 73.0	184.4 ± 85.8	> 0.999	238.6 ± 123.0	183.5 ± 99.8	> 0.999
paO2 / FiO2^2^	239.2 ± 85.0	214.7 ± 95.4	> 0.999	294.0 ± 96.1	223.9 ± 124.9	0.270
Hb [mg/dl]^2^	12.3 ± 1.1	11.5 ± 1.4	0.142	10.9 ± 1.4	11.8 ± 1.4	0.351
Lactate [mmol/l]^2^	0.77 ± 0.23	0.84 ± 0.29	> 0.999	0.81 ± 0.22	1.1 ± 0.53	0.396
BE^2^	-5.19 ± 2.2	-2.80 ± 2.4	**< 0.001**	-4.6 ± 1.6	-1.8 ± 3.0	**0.002**

^1^intra-individual mean ± SD; ^2^ inter-individual mean ± SD; ^3^ Wilcoxon Rank Sum tests and t-tests were used as appropriate, *P*-value are Bonferroni corrected; Haemodynamic and ventilation parameters were recorded every 15 min. ^4^ ΔP is the difference between Pplateau and PEEP in I-VATS patients, whereas Pmean– PEEP and Ppeak– PEEP is the pressure support in SV-VATS patients

Abbreviations: BP– blood pressure, MAP– mean arterial pressure, TV– tidal volume, PEEP– positive end-expiratory pressure, Pmean– mean inspiratory pressure, Ppeak– peak inspiratory pressure, Hb– haemoglobin, BE– base excess

### Respiratory parameters

In the group of anatomical and non-anatomical resections, SV-VATS patients received a significantly lower positive ed-expiratory pressure (PEEP) compared to I-VATS patients (both *P* < 0.001) (Table 4).

Anatomical SV-VATS patients showed a peak inspiratory pressure (Ppeak) of 7.2 ± 1.9 mbar and an inspiratory mean pressure (Pmean) of 4.2 ± 1.1 mbar. Anatomical I-VATS patients were subjected to an inspiratory plateau pressure (Pplateau) of 17.7 ± 2.9 mbar, resulting in a ΔP (i.e., Pplateau– PEEP) of 11.9 ± 1.4 mbar. Thus, the pressure support of anatomical SV-VATS patients, either calculated as Ppeak– PEEP (4.3 ± 1.9 mbar) or as Pmean– PEEP (1.2 ± 1.0 mbar), was significantly lower than ΔP of anatomical I-VATS patients (both *P* < 0.001) (Table 4).

Non-anatomical SV-VATS patients showed a Ppeak of 8.7 ± 3.2 mbar and a Pmean of 4.5 ± 1.3 mbar. Non-anatomical I-VATS patients were subjected to Pplateau of 17.6 ± 3.7 mbar, resulting in a ΔP of 11.6 ± 1.6 mbar. Hence, the pressure support of non-anatomical SV-VATS patients, either calculated as Ppeak– PEEP (5.3 ± 1.6 mbar) or a Pmean– PEEP (1.1 ± 0.5 mbar), was significantly lower than ΔP of non-anatomical I-VATS patients (both *P* < 0.001) (Table 4).

In the group of anatomical resections, patients with SV-VATS had significantly higher mean etCO2 (*P* < 0.001) and significantly lower SpO2 values (*P* = 0.003) during surgery compared to I-VATS patients (Table 4). Additionally, the coefficients of variation of the ventilation frequency (*P* < 0.001), the etCO2 (*P* < 0.001), and the tidal volume (TV) (*P* < 0.001) were significantly greater for SV-VATS patients. Thus, these parameters are more variable in SV-VATS patients. In the group of non-anatomical resections, patients with SV-VATS had significantly higher mean etCO2 values during surgery compared to I-VATS patients (*P* < 0.001). Additionally, the coefficient of variation of the ventilation frequency (*P* < 0.001) was significantly greater for SV-VATS patients, indicating a greater variability than for I-VATS patients. There was no significant difference between the respiratory parameters of I-VATS patients with and without volatile anaesthetic in addition to propofol (Suppl. Table S2).

### Blood gas analysis

In anatomical and non-anatomical resection, SV-VATS patients had a significantly lower pH (*P* < 0.001), significantly higher paCO2 (*P* < 0.001), and significantly more negative base excess (BE) (*P* < 0.001 for anatomical and *P* = 0.002 for non-anatomical resection) compared to I-VATS patients. The paO2/FiO2 quotient was not significantly different in SV- and I-VATS patients in either group (Table 4). In non-anatomical I-VATS patients receiving propofol, the BE was significantly more negative than I-VATS patients receiving propofol and volatile anaesthetic. There was no significant difference between further blood gas test parameters of I-VATS patients with and without desflurane or sevoflurane in addition to propofol (Suppl. Table S2). Respiratory acidosis and hypercarbia in SV-VATS patients was fully reversible (pH 7.37 ± 0.1 and paCO2 42.0 ± 9.8 mmHg) after the end of the operation.

### Anaesthetic processing times

**Fig. 3:**
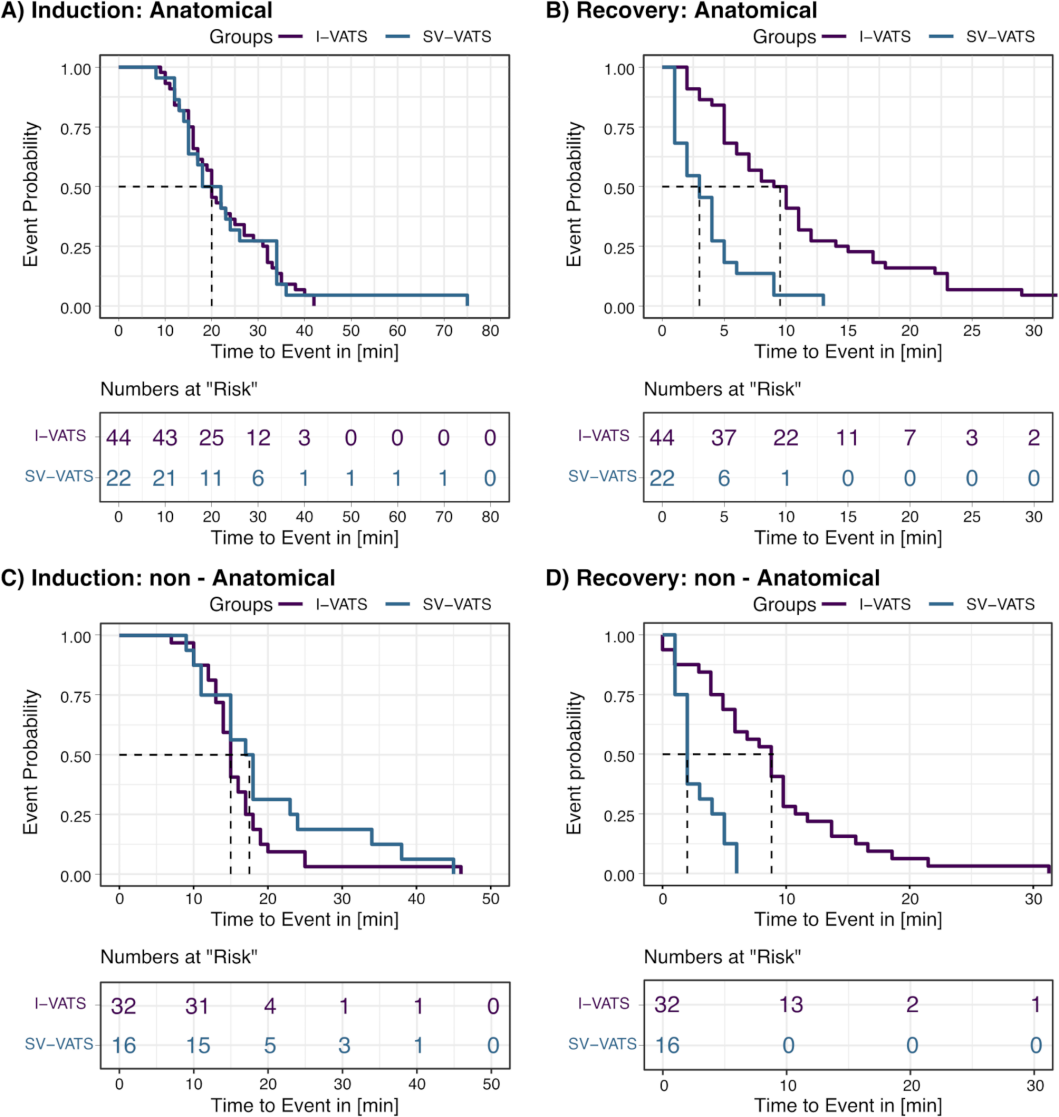
Kaplan-Meier plots for induction and recovery time for patients receiving anatomical resection (**A** and **B**) and for patients receiving non-anatomical resections (**C** and **D**). Dashed lines indicate the median time to event for either group. The lower panels of each sub-figure show the number of patients where the event had not yet occurred

For anatomical resections, the median time for induction of anaesthesia was 20 min in both groups (SV-VATS: IQR = 15.0–32.0; I-VATS: IQR = 15.75–31.25) and hence, not significantly different (*P* = 0.862, logrank test) (Fig. 3). The median incision-suture time was 107 min (IQR = 88–142.5) for SV-VATS patients and, thus, significantly shorter than in the I-VATS group (Median = 126 min; IQR = 109–171.25, *P* = 0.015, logrank test). The incision-suture time did not significantly correlate to the coefficient of correlations of propofol (r_adj_. = -0.22, *P* = 0.132), adjusted for the three groups SV-VATS, I-VATS with and without propofol, nor with the coefficient of correlations of remifentanil in the SV-VATS group (*r* = 0.12, *P* = 0.595).

The median time of recovery from anaesthesia was 3 min for SV-VATS (IQR = 1.0–4.75) and significantly (*P* < 0.001, logrank test) shorter than I-VATS by 6.5 min (Median = 9.5 min; IQR = 5.0–14.25) (Fig. 3). Time to recovery was not significantly affected by the incision-suture time (Cox-PH model: β = -0.005, LRT *P* = 0.063).

For non-anatomical resections, the median time for induction was 18 min for SV-VATS and 15 min for I-VATS patients and not significantly different (SV-VATS: IQR = 14.0–23.23, I-VATS: IQR = 13.0–17.25; *P* = 0.230, logrank test) (Fig. 3). The median incision-suture time was 44.5 min (IQR = 31–57.75) for SV-VATS patients, and not significantly different than in the I-VATS group (Median = 54 min; IQR = 47–88, *P* = 0.160, logrank test). The incision-suture time did not significantly correlate to the coefficient of correlations of propofol (r_adj_. = -0.13, *P* = 0.298), adjusted for the three groups SV-VATS, I-VATS with and without propofol, nor with the coefficient of correlations of remifentanil in the SV-VATS group (*r* = 0.05, *P* = 0.854).

The median time to recovery from anaesthesia was 2 min for SV-VATS (IQR = 1.75–4.25) and significantly (*P* < 0.001, logrank test) shorter than time to recovery from anaesthesia in I-VATS patients by 7 min (Median = 9 min; IQR = 4.75–11.25) (Fig. 3). Time to recovery was not significantly affected by the incision-suture time (Cox-PH model: b = 0.001, LRT *P* = 0.837).

### DLT selection

We analysed the size of the double-lumen tubes (DLT) the SV-VATS patients would have received by applying the “≥1 mm criterion” to a 3D reconstruction of the left main bronchus. The “≥1 mm criterion” stipulates at least a 1 mm difference between the patient’s left bronchial diameter and DLT’s outer diameter of the bronchial branch [11]. Table 5 compares the suggested DLT sizes to the ones selected by common selection methods based on demographic variables, i.e. sex and body height [29], based on the tracheal diameter in a chest X-ray [30], based on the bronchial diameter in a 2D chest CT [31] (Table 5). These methods suggest too large DLT sizes in 23–31% of the patients in reference to the ≥ 1 mm criterion.

**Table 5:** Suggested double-lumen tubes for patients receiving SV-VATS by current selection methods and by the ≥ 1 mm criterion and Mallinckrodt DLTs. The ≥ 1 mm criterion stipulates at least a 1 mm difference between the patient’s left bronchial diameter and DLT’s outer diameter of the bronchial branch [11]

Size [Fr]	Demographic, (29)	Chest X-Ray, (30)	2D-CT, (31)	≥ 1 mm Criterion, (11)
32^1^	0/38 (0.0%)	0/38 (0.0%)	0/38 (0.0%)	2/35 (5.7%)
35^1^	4/38 (10.5%)	6/38 (15.8%)	1/38 (2.6%)	7/35 (20.0%)
37^1^	18/38 (47.4%)	8/38 (21.1%)	3/38 (7.9%)	2/35 (5.7%)
39^1^	3/38 (7.9%)	9/38 (23.7%)	9/38 (23.7%)	0/35 (0.0%)
41^1^	13/38 (34.2%)	15/38 (39.5%)	25/38 (64.8%)	23/35 (65.7%)

^1^*n/N* (%); ^2^ For three patients 3D-reconstruction was not possible

Numbers may not add up to 100% due to rounding effects

**Fig. 4:**
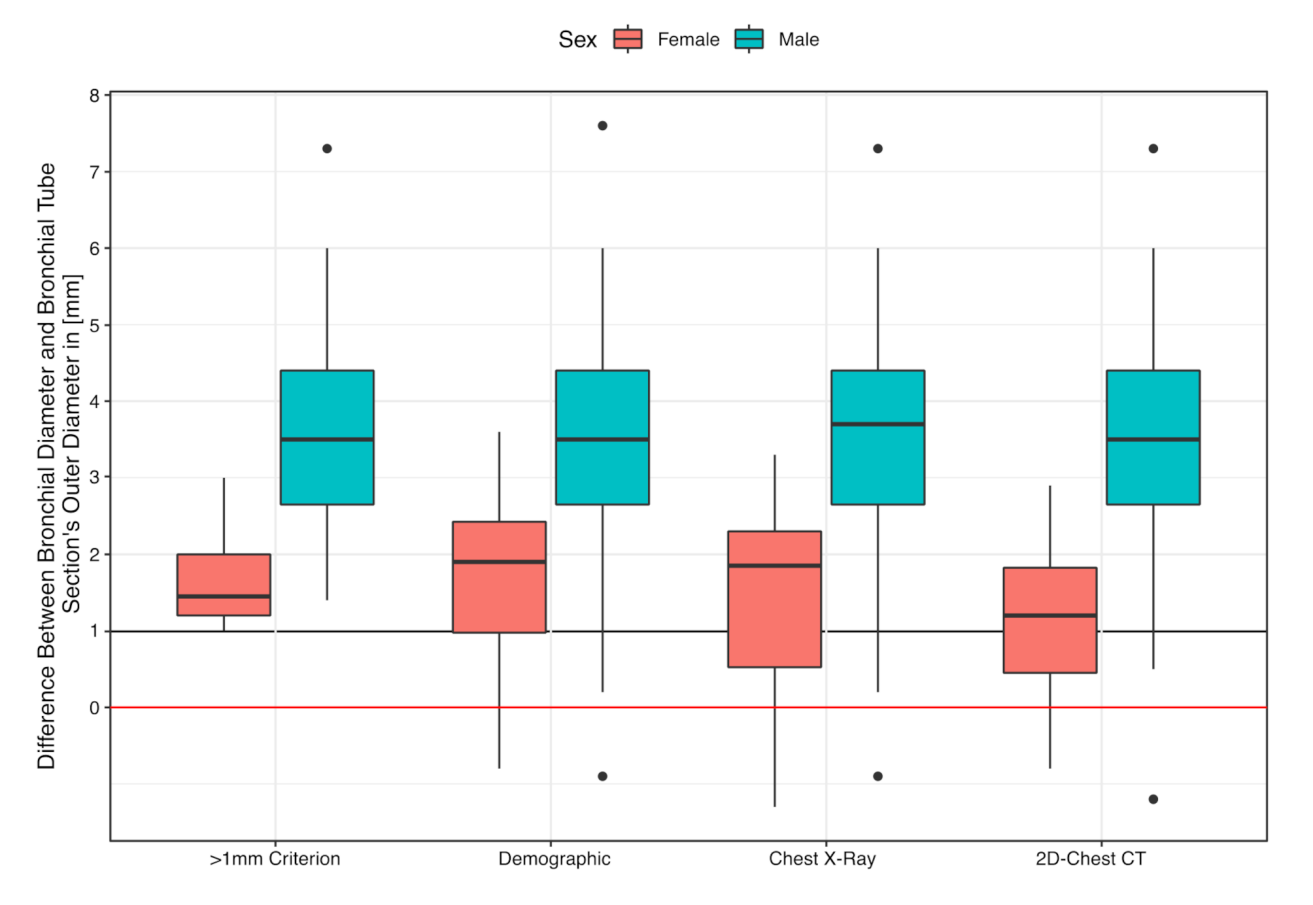
Box plots showing the difference between the left bronchial diameter and the bronchial tube section’s outer diameter for the “≥ 1 mm criterion” and the demographic method by Slinger et al., the method by Brodsky et al. based on the tracheal diameter on a chest X-ray, and the method by Hannallah et al. based on the bronchial diameter on a 2D-chest CT for male and female patients

3D-reconstruction of SV-VATS patients’ airways revealed that the difference between the diameter of the left main bronchus and the DLT’s outer bronchial diameter was below 1 mm in 25–40% of the female and 13% of the male patients, depending on the method used. More concerning, common selection methods [29–31] would have chosen a DLT size even greater than the patient’s bronchial diameter in 5–10% of the female and 5% of the male patients, depending on the method used (Fig. 4).

## Discussion

SV-VATS has been advocated to reduce pulmonary and airway complications during and after lung cancer surgery and to allow for faster postoperative recovery [9, 14, 15, 19, 33, 34]. However, evidence from prospective multicentre randomised trials to substantiate this view is still lacking. Our study provides data on standardised anaesthetic management of SV-VATS for the first time. It offers a prospective in-depth analysis of the haemodynamic, ventilation, blood gas test, and drug dosage of SV-VATS compared to matched I-VATS patients. Additionally, this analysis quantified the risk reduction using laryngeal masks instead of endobronchial DLT intubation.

SV-VATS leads to a higher variability in respiratory parameters than I-VATS without compromising hemodynamic stability. The data provides evidence that driving pressure, as well as tidal volumes during SV-VATS, are compatible with reduced mechanical power as compared to matched patients undergoing I-VATS. Lung protection was achieved by allowing permissive hypercapnia and respiratory acidosis without impairment of hemodynamic stability [35]. Hypercarbia and respiratory acidosis with values like those observed in our matched pairs study have been demonstrated to exert beneficial therapeutic effects during one-lung pressure ventilation with DLT [36].

Drug application by target control infusion of propofol (Marsh-model) and remifentanil (Minto-model), respectively, allowed for spontaneous ventilation during the entire operation. As compared to I-VATS, drug dosing was substantially reduced, and surgical access could be guaranteed even in the absence of muscle relaxation and OLV. However, the variability of drug dosing was much higher, reflecting continuous adjustment of drug dosing to secure both surgical access and spontaneous breathing efforts of the patients. Lower drug dosing is reflected in significantly higher BIS values in SV-VATS as compared to I-VATS patients. Our data provides the first detailed quantitative analysis of a defined anaesthetic management for SV-VATS, offering a rational basis for further risk evaluation of this anaesthetic concept.

Anaesthetic induction times were identical between SV-VATS and I-VATS patients, whereas the time from the end of the operation to the end of anaesthesia was significantly shorter in SV-VATS patients. The shorter time interval from the end of the operation to the end of anaesthesia most likely results from a lower depth of anaesthesia and the omission of muscle relaxation. Induction and recovery time are comparable to those reported in the literature [37–42]. Unlike several previous reports [5, 17, 43–53], however, we did not identify any changes in anaesthetic processing time by SV-VATS that would allow for a higher caseload in a real-world scenario of lung cancer resection. Given the duration of the operation and change times between operations, a reduction of anaesthetic processing time by 7 min would not allow treatment of more SV-VATS than I-VATS cases undergoing lung cancer resection during regular working hours.

Additionally, we analysed the DLT sizes SV-VATS patients may have received if being subjected to I-VATS instead. Current selection methods would have chosen DLT sizes that were too large in up to 40% of the patients, depending on the selection method used. We have recently demonstrated that all three methods [29–31] suffer from the fact that they predict the diameter of the left main bronchus inaccurately [11]. DLT selection remains problematic as without 3D reconstruction of the airway, there is no current method to accurately determine the left main bronchus, leading to tube size mismatch. Women are particularly at risk from oversized tubes and men from undersized tubes. The lack of an industry norm for DLTs makes choosing the correct DLT size all the more complex [11]. Thus, our analysis quantified the risk reduction by using laryngeal masks instead of DLTs in SV-VATS. Additionally, one could speculate that while face masks offer similar benefits in terms of ventilation and lung mechanics [16], laryngeal masks, as in SV-VATS, are likely safer and more effective in managing and responding to adverse situations compared to simple face masks during anaesthesia. Conversion to intubation remains a key consideration in SV-VATS, with reported rates ranging from 3% to 15% [9, 14]. Common reasons include insufficient lung collapse, hypoxemia, excessive coughing, or bleeding. While conversion may prolong operative time and increase complications, early recognition and standardised protocols help mitigate risks [22]. Further studies should consider predictors of conversion to optimise patient selection and improve SV-VATS safety [54, 55].

This study has several limitations. Firstly, though the group of SV-VATS patients was assessed prospectively, the group of I-VATS patients was matched from retrospective data. This means our study does not reach the quality of a prospective multicentre randomised study. The retrospective nature may introduce another potential bias, especially for variables that are not matched and, thus, not controlled for. Secondly, the study groups, especially when divided into anatomical and non-anatomical resections, are small and sampled in a single-centre setting, which limits generalizability. Thirdly, our study was not controlled for surgical aspects of the procedure. The group of patients was heterogeneous concerning surgical access, the amount of lung tissue removed, or the duration of the surgery, which may limit the generalizability. Differentiating between anatomical and non-anatomical resection may still be too coarse to reflect, especially, the surgical aspects. Surgical information, known prior to surgery, that may influence the decision on the anaesthesiologic procedure, such as the extent of resection, tumour size/location, prior thoracic surgery, pleural adhesions, uniportal or multiportal access, neoadjuvant therapy, comorbidities, smoking and others could also influence outcomes and should be deployed in larger prospective studies. The significant differences we found in the incision-suture times in the anatomical group of patients support this notion of unmatched surgical aspects. Also, anaesthesiologic aspects differed since some I-VATS patients received only propofol and others propofol and volatile anaesthetic without lidocaine inhalation and pharyngeal installation. Furthermore, SV-VATS patients received remifentanil and no relaxation, whereas I-VATS patients received fentanyl and muscle relaxation. However, this most probably would not change the interpretation of our results since propofol TCI in combination with remifentanil and no muscle relaxation would favour SV-VATS, whereas lidocaine inhalation in SV-VATS would favour I-VATS in the comparison of the processing times. Consequently, changing the anaesthetic drug regime in the I-VATS would rather decrease the difference in recovery times, making a clinically significant difference between both groups even more unlikely. Nevertheless, future studies aiming at establishing a difference in postoperative pulmonary complications between I-VATS and SV-VATS need to consider potentially confounding factors such as the beneficial effects of sevoflurane on the incidence and severity of postoperative pulmonary complications [56] and the adverse effect of muscle relaxation on (postoperative) inspiratory muscle strength [57] and its decreasing effect on the sensitivity to hypoxia [58]. Finally, another limitation of our study is the reduction in sample size due to PSM. As only 44 of 95 anatomical I-VATS patients and 32 of 92 non-anatomical I-VATS patients were matched, our findings cannot be directly generalised to the entire population of I-VATS patients. This limitation is inherent to PSM, as the method excludes cases that do not have a close match, thereby improving internal validity but reducing external validity. Furthermore, PSM only adjusts for measured confounders, leaving potential residual bias from unmeasured variables [59].

This study has several advantages: Our study leverages a matched pairs analysis, providing robust control over potential confounding variables. Matching patients on 13 preoperative covariates ensures that outcome differences between SV-VATS and I-VATS are more likely due to surgical and anaesthetic techniques rather than patient characteristics or other patient characteristics beyond these 13 covariates. Critical anaesthetic variables such as depth of anaesthesia, hypercarbia, and respiratory settings are carefully controlled by minimising potential confounding effects, allowing for a more accurate comparison between the two techniques. Since moderate hypercarbia is one of the key differences between SV-VATS and I-VATS, which we and others observed [15], our analysis is advantaged by not only leveraging a prospective SV-VATS group but also by matching I-VATS patients based on the pre-operative lung function. Our results highlight the need for future studies, especially randomised trials, to standardise not only the management of these factors to avoid bias but also lung function as an inclusion criterion. In summary, the methodological rigour of our study design strengthens the validity of our findings and provides a solid foundation for future research in the field.

Our study provides the first data-driven analysis of haemodynamic, ventilation, blood gas analyses, and medication dosage in standardised anaesthetic management of SV-VATS by a matched pair comparison to I-VATS. Our data confirms that SV-VATS is feasible for a wide variety of operations in lung cancer surgery. Respiratory acidosis seems inherent to the method and is well tolerated and reversible. Oxygenation and hemodynamic stability are not different from I-VATS, with SV-VATS providing less invasive respiratory support. As compared to I-VATS, adjustment of anaesthetic drug dosing is frequently necessary for SV-VATS to keep the patient spontaneously breathing and, at the same time, allow for surgical access. Allowing for the same respiratory acidosis in I-VATS patients as in SV-VATS patients would most likely reduce the invasiveness of ventilation in I-VATS patients as well [35]. In I-VATS, it may thus be worth aiming at a reduction of mechanical power rather than normocapnia and a balanced pH [60–62]. Future trials must reflect this in their design and the possible risk imposed by the necessity to convert to tracheal intubation during the operation [16, 63].

Taken together, the results of this matched pairs analysis suggest that establishing the beneficial effects of and the role of SV-VATS in non-anatomical and anatomical resection for lung cancer surgery needs well-standardized and randomised prospective multicentre outcome trials, taking into account several confounding factors, including anaesthetic factors. Studies comparing SV-VATS and I-VATS need to be controlled not only for surgical but also for anaesthetic management, including blood gases. Since previous studies emphasised that complication rates and beneficial effects are significantly influenced by local institutional practices rather than the anaesthesia method alone, future studies should focus on standardised perioperative protocols and prospective randomised trials to isolate the true impact of SV-VATS [9, 14, 22]. In the meantime, individual patients may be identified as benefiting from standardised perioperative treatment concepts of SV-VATS, such as those described in this study. In addition, it may be worth considering less invasive ventilation in I-VATS patients, trading in normocarbia and a balanced pH for reduced mechanical power during one-lung ventilation [60–62].

## Conclusions

Our study provides the first prospective quantitative in-depth analysis of a standardised anaesthetic management regime for SV-VATS, including anaesthetic processing times. Respiratory parameters during SV-VATS are compatible with reduced mechanical power as compared to patients undergoing I-VATS. The anaesthetic management regime reduced the risk of airway damage imposed by choosing too-large DLTs in up to 10% of patients without compromising oxygenation and hemodynamic stability. Changes in anaesthetic processing time by 7 min would not allow for a higher caseload of SV-VATS for lung cancer surgery.

## Electronic supplementary material

Below is the link to the electronic supplementary material.

Supplementary Material 1

Supplementary Material 2

## Data Availability

All data generated or analysed during this study are included in this published article and its supplementary information files.

## Abbreviations

BE: Base excess
BGA: Blood gas analysis
BMI: Body mass index
BP: Blood pressure
Cox-PH: Cox’s proportional hazard
CV: Coefficient of variation
DLCO-SB: Lung diffusion capacity for CO
DLT: Double-lumen tube
FEV1: Forced expiratory volume in 1 s
FRC: Functional residual capacity
FVC: Forced vital capacity
Hb: Haemoglobin
IQR: Inter-quantile range
I-VATS: Intubated video-assisted thoracoscopic surgery
LRT: Likelihood ratio test
MAP: Mean arterial pressure
MEF50: Maximal expiratory flow at 50% of FVC
NI-VATS: Non-intubated video-assisted thoracoscopic surgery
OLV: One-lung ventilation
PEEP: Positive end-expiratory pressure
PEF: Peak expiratory flow
Pmean: Mean inspiratory pressure
Ppeak: Peak inspiratory pressure
Pplateau: Inspiratory plateau pressure
PSM: Propensity score matching
RCRI: Revised cardiac risk index
SD: Standard deviation
SMD: Standardised mean difference
sRtot: Total specific resistance
SV-VATS: Spontaneous ventilation video-assisted thoracoscopic surgery
TCI: Target-controlled infusion
TLC: Total lung capacity
TV: Tidal volume
VA: Volatile anaesthetic
VATS: Video-assisted thoracoscopic surgery
VC: Vital capacity
