# Supplementary material for: Prospective in-depth analysis of anaesthetic management of spontaneous ventilation VATS for lung cancer resection: a matched pairs comparison to intubated VATS
Source: BMC Anesthesiol. 2025 Apr 16;25:185. doi: 10.1186/s12871-025-03027-9 (PMC12004653; doi:10.1186/s12871-025-03027-9)
Supplement: Supplementary file 2 — Supplementary Material 2 [file 12871_2025_3027_MOESM2_ESM.docx]

# SUPPLEMENTARY TABLES

Supplementary Table S1. *Standardised mean differences (SMD) of pre-operative parameters between SV-VATS and I-VATS patients before and after propensity score matching (PSM). P-values are given for differences after matching between SV- and I-VATS groups.*

|  | **Anatomical** | | | **Non-Anatomical** | | |
| --- | --- | --- | --- | --- | --- | --- |
|  | **Before PSM** | **After PSM** | **P - Value** | **Before PSM** | **After PSM** | **P - Value** |
| Age | 0.323 | 0.103 | 1.000 | -0.265 | -0.155 | 0.582 |
| Sex | 0.379 | 0 | 0.714 | 0.038 | -0.063 | 1.000 |
| Height | -0.458 | -0.116 | 0.682 | 0.322 | 0.090 | 0.757 |
| Weight | -0.476 | -0.002 | 0.995 | 0.223 | -0.016 | 0.955 |
| BMI | -0.185 | 0.064 | 0.846 | 0.049 | -0.092 | 0.752 |
| FVC | -0.237 | -0.116 | 0.726 | 0.421 | 0.150 | 0.596 |
| FEV1 | -0.040 | -0.101 | 0.746 | 0.417 | 0.137 | 0.587 |
| FVC / FEV1 | 0.764 | 0.125 | 0.741 | 0.271 | -0.078 | 0.821 |
| TLC | -0.611 | -0.095 | 0.743 | 0.392 | 0.163 | 0.602 |
| DLCO SB | -0.991 | 0.031 | 0.929 | - | - | - |
| paO2 | 0.325 | -0.002 | 0.994 | 0.216 | -0.039 | 0.891 |
| paCO2 | 0.63 | 0.121 | 0.662 | 0.001 | 0.236 | 0.455 |
| Haemoglobin | -0.334 | -0.109 | 0.739 | -0.786 | 0.171 | 0.569 |
| **Total** | 0.854 | 0.265 | - | 1.014 | 0.103 | - |

**Supplementary Table S2.** *Intra-individual means and SD of intra-operative parameters are compared between I-VATS patients with propofol and with or without volatile anaesthetic. P-values are Bonferroni corrected.*

|  | **I-VATS – Anatomical Resections** | | | **I-VATS – Non-Anatomical Resections** | | |
| --- | --- | --- | --- | --- | --- | --- |
|  | **Propofol**, N = 8 | **Propofol+VA**^3^, N = 36 | **P – Value**^4^ | **Propofol**, N = 7 | **Propofol+VA**^3^, N = 25 | **P – Value**^4^ |
| **Hemodynamic Variables** | | | | | | |
| Heart Rate [1/min]^1^ | 68.0 ± 14.1 | 62.1 ± 8.0 | 0.892 | 60.6 ± 7.1 | 67.4 ± 13.1 | 0.896 |
| Syst. BP [mmHg]^1^ | 111.8 ± 14.6 | 112.4 ± 10.1 | >0.999 | 112.7 ± 11.5 | 108.5 ± 10.6 | >0.999 |
| Dia. BP [mmHg]^1^ | 60.2 ± 8.0 | 58.5 ± 6.1 | >0.999 | 61.7 ± 4.7 | 59.0 ± 4.5 | 0.700 |
| MAP [mmHg]^1^ | 77.4 ± 9.2 | 76.5 ± 6.8 | >0.999 | 78.7 ± 6.2 | 75.5 ± 6.0 | 0.764 |
| **Ventilation Setting** | | | | | | |
| Vent. Freq. [1/min]^1^ | 13.7 ± 2.8 | 14.1 ± 1.2 | >0.999 | 13.8 ± 1.3 | 13.8 ± 1.6 | >0.999 |
| etCO2 [mmHg]^1^ | 35.5 ± 6.8 | 35.3 ± 3.7 | >0.999 | 32.9 ± 3.5 | 35.0 ± 4.7 | >0.999 |
| SpO2 [%]^1^ | 98.8 ± 2.2 | 98.9 ± 1.1 | >0.999 | 98.8 ± 1.3 | 98.3 ± 1.9 | >0.999 |
| TV [ml/kg_PBW_]^1^ | 5.4 ± 1.0 | 5.9 ± 1.9 | >0.999 | 5.3 ± 1.0 | 5.4 ± 1.2 | >0.999 |
| PEEP [mbar]^1^ | 5.7 ± 2.0 | 5.8 ± 1.1 | >0.999 | 6.0 ± 1.1 | 6.1 ± 0.9 | >0.999 |
| Pplateau [mbar]^1^ | 19.0 ± 3.3 | 17.4 ± 2.6 | >0.999 | 17.7 ± 1.8 | 17.6 ± 3.2 | >0.999 |
| ΔP [mbar]^1^ | 13.3 ± 3.1 | 11.6 ± 2.5 | 0.400 | 11.6 ± 1.7 | 11.5 ± 3.7 | >0.999 |
| **Blood Gas Tests** | | | | | | |
| pH^2^ | 7.3 ± 0.1 | 7.4 ± 0.1 | >0.999 | 7.4 ± 0.00 | 7.4 ± 0.02 | >0.999 |
| paCO2 [mmHg]^2^ | 44.5 ± 13.7 | 40.2 ± 6.4 | >0.999 | 37.4 ± 7.7 | 38.8 ± 5.0 | >0.999 |
| paO2 [mmHg]^2^ | 177.5 ± 103.9 | 185.9 ± 79.5 | >0.999 | 137.9 ± 89.9 | 191.8 ± 95.7 | >0.999 |
| FiO2 [%]^2^ | 84.2 ± 1.94 | 86.2 ± 1.18 | >0.999 | 78.4 ± 2.3 | 86.5 ± 1.8 | >0.999 |
| paO2 / FiO2^2^ | 207.2 ± 98.4 | 216.4 ± 85.0 | >0.999 | 137.9 ± 89.9 | 239.6 ± 118.3 | >0.999 |
| Hb [mg/dl]^2^ | 11.8 ± 1.2 | 11.5 ± 1.3 | >0.999 | 11.9 ± 0.3 | 10.7 ± 1.5 | 0.312 |
| Lactate [mmol/l]^2^ | 0.82 ± 0.19 | 0.85 ± 0.26 | >0.999 | 1.5 ± 0.11 | 1.0 ± 0.3 | >0.999 |
| BE^2^ | -3.24 ± 0.9 | -2.70 ± 2.2 | >0.999 | -2.5 ± 2.9 | -1.7 ± 3.1 | **0.016** |
| ^1^intra-individual mean±SD;  ^2^ inter-individual mean±SD;  ^3^ VA – volatile anaesthetic (sevoflurane or desflurane);  ^4^ Wilcoxon Rank Sum tests and t-tests were used as appropriate.  P-values are Bonferroni corrected; Haemodynamic and ventilation parameters were recorded every 15 minutes. | | | | | | |
